# Trends in Medical Expenses for Ocular Examinations in Japan Based on Nationwide Database Studies

**DOI:** 10.31662/jmaj.2025-0216

**Published:** 2025-09-19

**Authors:** Tomoko Kanaya

**Affiliations:** 1 Department of Social Medicine, Asahikawa Medical University, Asahikawa, Japan

**Keywords:** medical expenses, Japan, contact lens, optical coherence tomography

## Abstract

**Introduction:**

Medical expenses, including ocular expenses, have been increasing annually in Japan. This study investigated national trends in the number of ocular examinations and examination expenses.

**Methods:**

This descriptive study used two national data sources: national medical expense estimates and statistics on medical care activities under public health insurance. We analyzed the medical expenses in Japan from 2008 to 2021 and estimated the proportions of different types of medical services.

**Results:**

Over 14 years, total medical expenses increased from 1.71 trillion yen to 2.31 trillion yen, with ocular medical expenses increasing from 64.5 billion yen to 96.0 billion yen. The total examination expenses increased from 155.2 billion yen to 231.5 billion yen, with ocular examination expenses increasing from 24.3 billion yen to 32.8 billion yen over 14 years. The examination expense category accounted for 32.3% of ocular medical expenses, although the overall examination expense category amounted to 10.0% of nationwide medical expenses in 2021. In particular, expenses related to optical coherence tomography (OCT) surged by 875.6%, visual field testing increased by 81.2%, corneal curvature measurement increased by 61.0%, and corneal endothelial cell density measurement increased by 59.3%. Only contact lens (CL) examination fees expenses decreased by 10.9%. In 2020, the coronavirus disease 2019 (COVID-19) pandemic caused a 24.8% and 22.0% decrease in ocular and nationwide examination expenses, respectively.

**Conclusions:**

Over 14 years, ocular medical expenses in Japan have increased by 49.0%, and nationwide medical expenses have increased by 35.1%. In addition, ocular examination expenses have increased by 35.0% over the past 14 years, although nationwide examination expenses have increased by 49.1%. All examination expenses increased except for CL examination fees. In particular, the OCT category witnessed a rapid increase. The COVID-19 pandemic has affected ocular examination expenses.

## Introduction

Investigating trends in national healthcare and comprehending the current state of healthcare services are crucial. Medical expenses in each department may increase with medical expenses. Visual information is the primary source of information that we receive in our daily lives, and it is crucial to protect our eyes to maintain our quality of life.

Previous studies on ophthalmology expenses in Japan have focused on major diseases, such as cataracts, glaucoma, vitreous retinal diseases ^(1), (2), (3), (4)^, economic losses due to ophthalmic diseases (primarily visual impairment) ^(5), (6)^, the expenses of surgery and medications ^(7), (8), (9)^, and trends in ocular examination expenses have not been investigated.

Ocular examinations are directly linked to the diagnostic results and treatment plans. Understanding the trends in ocular examination expenses is useful for formulating medical fees in the ophthalmology field. Ocular examination equipment has advanced, and the variety of available tests has increased; therefore, the need for examinations in daily practice may have changed in recent years. In addition, the coronavirus disease 2019 (COVID-19) pandemic has possibly restricted ocular medical and examination expenses.

In this study, we analyzed the national big data provided by the Ministry of Health, Labor, and Welfare (MHLW), which records the medical procedures of 125.5 million citizens. The purpose of this study was to assess medical treatment expenses in Japan in 2021 and clarify the trends in ocular examination expenses in Japan from 2008 to 2021.

## Materials and Methods

### Data sources

Japan has implemented a universal health insurance system. The MHLW records the detailed medical procedures nationwide and makes them available on its website. In this descriptive study, two national data sources were used: the Estimate of National Medical Care Expenditure (ENMCE) ^(10)^ and the Statistics of Medical Care Activities in Public Health Insurance (MCAPHI) ^(11)^ from the e-Stat on the MHLW website.

The ENMCE is an estimated medical expense covered by medical insurance treatment. National medical expenses cover expenses for medical services under the national health insurance system. The medical care system is limited to medical expenses for injuries and illnesses; it does not include expenses required for normal pregnancy and childbirth, health checkups and vaccinations, artificial eyes and limbs required for fixed physical disabilities, elective medical treatment (multifocal intraocular lens), and medical treatments not covered by the insurance system (contact lens [CL] and glasses).

National medical expenses were divided into medical treatment expenses, dental treatment expenses, pharmacy dispensing expenses, meals and living expenses during hospitalization, visiting nursing expenses, and medical expenses for treatment by judo therapists and acupuncturists. Because the statistical methods for medical treatment types were reorganized after 2008, this study examined data from 2008 to 2021.

In this study, we examined the annual total amount and average per capita expenses for total medical treatment expenses and those for eye and adnexal disease VII (International Classification of Diseases, Tenth Revision [ICD-10] [2013]-based injury and disease classification [H00-59]) from ENMCE.

The second data source, the MCAPHI, aimed to clarify the content of medical treatment procedures (medical and dental), dispensing procedures, and drug usage status. For medical benefit recipients under the medical insurance system, the number of times and points, and the status of the review are published every June. As expense data for each medical procedure were not available from the ENMCE, data on medical procedures (ICD-10) from the MCAPHI were used to calculate the percentages of medical expenses by medical procedure (initial and repeat consultation fee, medical management, at-home treatment, examinations, diagnostic imaging, drug administration, injection, rehabilitation, psychiatric specialty therapy, treatments, surgery, anesthesia, radiotherapy, pathological diagnosis, hospitalization fee, and Diagnosis Procedure Combination/Per-Diem Payment System [DPC/PDPS]).

We investigated the trends in the number of times and expenses for examinations performed nationwide in June of each year, focused on the examination category of ‘medical procedures (subclassification)’ from the MCAPHI.

Surcharges for ocular examinations (newborns, infants, and young children) were excluded. We were unable to survey newly established examinations due to the revision of medical fees for the years after 2008; therefore, we investigated the trends since their establishment.

Only open data sources were used in this study, and ethical review and approval were not required.

## Results

### Japan’s national health insurance system

In 2021, Japan had 125 million individuals, and medical expenses totaled 45 trillion yen, with a per capita expense of 358,800 yen. Medical expenses accounted for 8.18% of gross domestic product (550.5 trillion yen) ^(10)^. Specifically, the total cost of medical treatment annually was 32.4 trillion yen (including hospitalization expenses of 16.9 trillion yen (37.4%) and outpatient expenses of 15.5 trillion yen [34.5%]), which accounted for 70% of the total national medical expenses. Ocular medical expenses totaled 1,163 billion yen, with hospitalization costs accounting for 237 billion yen, and outpatient expenses accounting for 926 billion yen ^(10)^.

### National medical expenses in June

**Figure 1.**
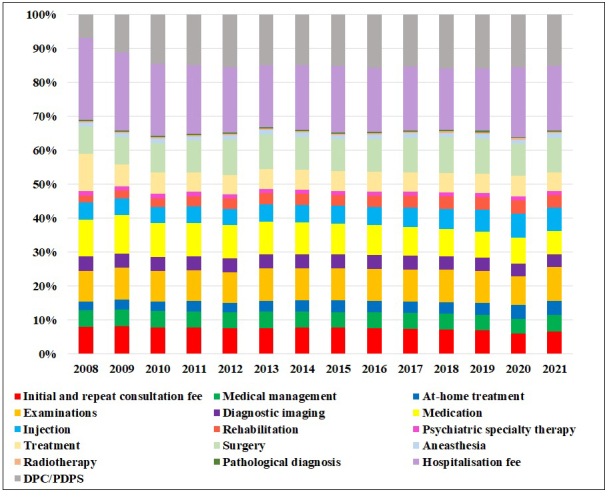
Trends in the proportion of services in medical expenses per capita for overall diseases in June 2008-2021.

Table 1 and Figure 1 show the total medical expenses and per capita expenditure trends for overall diseases from the MCAPHI. The average total medical expenditure for all diseases from June 2008 to June 2010 increased by 35.1% from 1.71 trillion yen to 2.31 trillion yen in 2021 (Table 1).

**Table 1.** Trends in the Proportion of Services in Medical Expenses Per Capita for Overall Diseases in June 2008-2021.

Year	Total medical expenses (1 trillion yen)	Total medical expenses per capita (yen)	Initial and repeat consultation fee (1 trillion yen), n, (%)	Initial and repeat consultation fee per capita (yen)	Medical management (1 trillion yen), n, (%)	Medical management per capita (yen)	At-home treatment (1 trillion yen), n, (%)	At-home treatment per capita (yen)	Examinations (1 trillion yen), n, (%)	Examinations per capita (yen)	Diagnostic imaging (1 trillion yen), n, (%)	Diagnostic imaging per capita (yen)
2008	1.74	13,603.7	0.140 (8.1)	1,097.7	0.086 (4.9)	670.7	0.043 (2.5)	340.2	0.154 (8.9)	1,208.2	0.076 (4.4)	592.6
2009	1.66	13,008.6	0.135 (8.1)	1,055.5	0.084 (5.0)	655.6	0.047 (2.8)	367.6	0.156 (9.4)	1,224.0	0.069 (4.2)	541.7
2010	1.73	13,471.7	0.136 (7.9)	1,059.4	0.085 (4.9)	663.4	0.047 (2.7)	369.9	0.155 (9.0)	1,212.5	0.069 (4.0)	537.0
Avg. in 2008-2010	1.71	13,361.3	0.137 (8.0)	1,070.8	0.085 (5.0)	663.2	0.046 (2.7)	359.2	0.155 (9.1)	1,214.9	0.071 (4.2)	557.1
2011	1.91	14,943.1	0.148 (7.8)	1,161.2	0.089 (4.7)	697.1	0.061 (3.2)	475.7	0.172 (9.0)	1,345.0	0.078 (4.1)	608.2
2012	1.99	15,639.8	0.151 (7.5)	1,180.8	0.094 (4.7)	739.2	0.057 (2.8)	445.1	0.179 (9.0)	1,405.6	0.080 (4.0)	627.6
2013	2.13	16,761.1	0.163 (7.7)	1,283.6	0.102 (4.8)	797.6	0.069 (3.2)	542.9	0.203 (9.5)	1,592.8	0.089 (4.2)	700.1
2014	2.14	16,823.8	0.168 (7.8)	1,319.8	0.102 (4.8)	799.2	0.068 (3.2)	537.5	0.203 (9.5)	1,601.2	0.089 (4.2)	698.3
2015	2.14	16,821.4	0.166 (7.8)	1,304.5	0.099 (4.6)	782.1	0.071 (3.3)	561.3	0.202 (9.5)	1,591.5	0.087 (4.1)	684.7
2016	2.18	17,208.8	0.166 (7.6)	1,304.0	0.102 (4.7)	803.3	0.073 (3.3)	573.4	0.208 (9.5)	1,638.3	0.089 (4.1)	703.8
2017	2.30	18,119.0	0.172 (7.5)	1,354.5	0.105 (4.6)	829.8	0.076 (3.3)	603.1	0.217 (9.5)	1,715.7	0.094 (4.1)	743.4
2018	2.34	18,491.5	0.170 (7.3)	1,345.5	0.107 (4.6)	844.1	0.080 (3.4)	633.4	0.222 (9.5)	1,756.2	0.096 (4.1)	758.9
2019	2.36	18,706.9	0.167 (7.1)	1,319.8	0.105 (4.5)	834.6	0.083 (3.5)	656.1	0.222 (9.4)	1,759.4	0.095 (4.0)	754.1
2020	2.03	16,119.8	0.121 (6.0)	959.7	0.089 (4.4)	703.3	0.086 (4.2)	679.4	0.172 (8.5)	1,362.6	0.073 (3.6)	577.9
2021	2.31	18,380.5	0.152 (6.6)	1,207.8	0.115 (5.0)	912.7	0.093 (4.0)	742.2	0.231 (10.0)	1,844.3	0.086 (3.7)	682.0
**Year**	**Medication (1 trillion yen), n, (%)**	**Medication per capita (yen)**	**Injection (1 trillion yen), n, (%)**	**Injection per capita (yen)**	**Rehabilitation (1 trillion yen), n, (%)**	**Rehabilitation per capita (yen)**	**Psychiatric specialty therapy (1 trillion yen), n, (%)**	**Psychiatric specialty therapy per capita (yen)**	**Treatment (1 trillion yen), n, (%)**	**Treatment per capita (yen)**	**Surgery (1 trillion yen), n, (%)**	**Surgery per capita (yen)**
2008	0.187 (10.7)	1,461.8	0.089 (5.1)	693.3	0.036 (2.1)	280.4	0.024 (1.4)	189.0	0.188 (10.8)	1,475.9	0.140 (8.0)	1,093.3
2009	0.190 (11.5)	1,492.5	0.081 (4.9)	631.5	0.037 (2.2)	291.5	0.021 (1.3)	167.2	0.105 (6.3)	823.7	0.134 (8.1)	1,047.7
2010	0.174 (10.1)	1,361.2	0.079 (4.6)	615.0	0.045 (2.6)	352.7	0.025 (1.4)	192.0	0.108 (6.3)	846.9	0.148 (8.6)	1,157.0
Avg. in 2008-2010	0.184 (10.8)	1,438.5	0.083 (4.8)	646.6	0.039 (2.3)	308.2	0.023 (1.4)	182.7	0.134 (7.8)	1,048.8	0.140 (8.2)	1,099.3
2011	0.190 (10.0)	1,490.3	0.093 (4.9)	728.3	0.055 (2.9)	429.9	0.025 (1.3)	197.7	0.108 (5.7)	848.1	0.179 (9.4)	1,403.1
2012	0.195 (9.8)	1,528.7	0.096 (4.8)	749.4	0.062 (3.1)	485.4	0.023 (1.2)	180.4	0.114 (5.7)	890.7	0.206 (10.4)	1,619.0
2013	0.206 (9.6)	1,617.2	0.109 (5.1)	858.7	0.069 (3.2)	542.5	0.025 (1.2)	198.5	0.126 (5.9)	986.5	0.217 (10.2)	1,702.7
2014	0.198 (9.3)	1,561.6	0.110 (5.2)	869.1	0.071 (3.3)	559.5	0.026 (1.2)	201.4	0.126 (5.9)	992.9	0.205 (9.6)	1,610.1
2015	0.195 (9.1)	1,532.0	0.112 (5.2)	878.4	0.070 (3.3)	553.0	0.025 (1.2)	197.8	0.125 (5.9)	986.7	0.198 (9.3)	1,560.1
2016	0.192 (8.8)	1,510.5	0.116 (5.3)	916.7	0.074 (3.4)	585.7	0.026 (1.2)	203.2	0.125 (5.7)	986.5	0.212 (9.7)	1,667.9
2017	0.195 (8.5)	1,538.2	0.129 (5.6)	1,018.2	0.080 (3.5)	634.1	0.027 (1.2)	215.1	0.132 (5.8)	1,045.7	0.234 (10.2)	1,844.0
2018	0.187 (8.0)	1,477.5	0.138 (5.9)	1,095.1	0.084 (3.6)	666.4	0.028 (1.2)	223.9	0.132 (5.6)	1,041.9	0.247 (10.6)	1,955.4
2019	0.181 (7.7)	1,431.8	0.153 (6.5)	1,209.9	0.084 (3.5)	663.5	0.028 (1.2)	220.4	0.135 (5.7)	1,073.3	0.248 (10.5)	1,965.3
2020	0.155 (7.6)	1,226.7	0.144 (7.1)	1,144.5	0.080 (3.9)	632.1	0.025 (1.2)	194.9	0.124 (6.1)	980.5	0.192 (9.5)	1,524.6
2021	0.158 (6.9)	1,262.7	0.159 (6.9)	1,266.4	0.084 (3.7)	672.8	0.027 (1.2)	215.5	0.127 (5.5)	1,013.8	0.238 (10.3)	1,892.8
**Year**	**Anesthesia (1 trillion yen), n, (%)**	**Anesthesia per capita (yen)**	**Radiotherapy (1 trillion yen), n, (%)**	**Radiotherapy per capita (yen)**	**Pathological diagnosis (1 trillion yen), n, (%)**	**Pathological diagnosis per capita (yen)**	**Hospitalization fee (1 trillion yen), n, (%)**	**Hospitalization fee per capita (yen)**	**DPC/PDPS (1 trillion yen), n, (%)**	**DPC/PDPS per capita (yen)**		
2008	0.023 (1.3)	177.6	0.004 (0.3)	35.2	0.007 (0.4)	55.9	0.420 (24.2)	3,292.9	0.120 (6.9)	938.8		
2009	0.021 (1.3)	168.0	0.004 (0.3)	34.8	0.007 (0.4)	54.6	0.380 (22.9)	2,978.0	0.188 (11.3)	1,475.0		
2010	0.024 (1.4)	187.8	0.006 (0.3)	47.1	0.008 (0.5)	60.9	0.364 (21.1)	2,845.9	0.251 (14.6)	1,963.1		
Avg. in 2008-2010	0.023 (1.3)	177.8	0.005 (0.3)	39.0	0.007 (0.4)	57.1	0.388 (22.7)	3,038.9	0.186 (10.9)	1,459.0		
2011	0.025 (1.3)	196.9	0.007 (0.4)	55.4	0.008 (0.4)	63.7	0.383 (20.1)	2,996.4	0.287 (15.0)	2,243.7		
2012	0.028 (1.4)	222.1	0.008 (0.4)	61.5	0.009 (0.4)	68.3	0.381 (19.1)	2,986.4	0.312 (15.6)	2,447.3		
2013	0.029 (1.4)	229.5	0.008 (0.4)	65.5	0.010 (0.5)	76.1	0.389 (18.2)	3,052.7	0.320 (15.0)	2,511.9		
2014	0.028 (1.3)	220.3	0.008 (0.4)	63.6	0.009 (0.4)	74.3	0.407 (19.0)	3,201.0	0.319 (14.9)	2,511.4		
2015	0.026 (1.2)	207.7	0.008 (0.4)	61.7	0.009 (0.4)	71.6	0.418 (19.6)	3,289.0	0.325 (15.2)	2,555.6		
2016	0.028 (1.3)	218.0	0.009 (0.4)	67.0	0.010 (0.4)	75.3	0.411 (18.8)	3,238.0	0.345 (15.8)	2,716.4		
2017	0.030 (1.3)	240.1	0.009 (0.4)	69.7	0.010 (0.4)	79.0	0.429 (18.7)	3,386.7	0.355 (15.5)	2,800.1		
2018	0.031 (1.3)	245.3	0.010 (0.4)	81.5	0.010 (0.4)	82.4	0.422 (18.1)	3,339.5	0.372 (15.9)	2,944.4		
2019	0.031 (1.3)	242.5	0.010 (0.4)	81.7	0.010 (0.4)	82.3	0.432 (18.3)	3,425.3	0.377 (16.0)	2,986.8		
2020	0.024 (1.2)	189.7	0.010 (0.5)	75.4	0.007 (0.4)	57.0	0.415 (20.4)	3,290.6	0.318 (15.6)	2,520.8		
2021	0.029 (1.3)	231.7	0.010 (0.4)	82.1	0.010 (0.4)	77.5	0.439 (19.0)	3,494.1	0.349 (15.1)	2,781.9		

Avg.: average; DPC/PDPS: Diagnosis Procedure Combination/Per-Diem Payment System.

**Figure 2.**
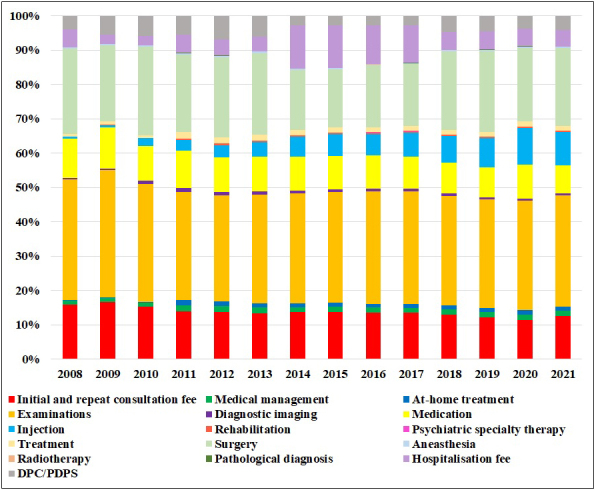
Trends in the proportion of services in medical expenses per capita for ophthalmic diseases in June 2008-2021.

Table 2 and Figure 2 display the total medical expenses for ophthalmic diseases (eye and adnexal disease VII) according to the MCAPHI and per capita expenditure trends. Medical expenses for ophthalmic diseases in June increased by 49.0%, from an average of 64.5 billion yen from 2008 to 2010 to 96.0 billion yen in 2021, a growth rate greater than that of total medical expenses (Table 2).

**Table 2.** Trends in the Proportion of Services in Medical Expenses Per Capita for Ophthalmic Diseases in June 2008-2021.

Year	Total medical expenses (1 billion yen)	Total medical expenses per capita (yen)	Initial and repeat consultation fee (1 billion yen), n, (%)	Initial and repeat consultation fee per capita (yen)	Medical management (1 billion yen), n, (%)	Medical management per capita (yen)	At-home treatment (1 billion yen), n, (%)	At-home treatment per capita (yen)	Examinations (1 billion yen), n, (%)	Examinations per capita (yen)	Diagnostic imaging (1 billion yen), n, (%)	Diagnostic imaging per capita (yen)
2008	66.2	518.2	10.5 (15.8)	81.8	0.8 (1.2)	6.2	0.1 (0.2)	0.9	23.2 (35.1)	181.9	0.3 (0.4)	2.0
2009	61.1	478.9	10.1 (16.5)	79.1	0.8 (1.3)	6.0	0.1 (0.2)	0.9	22.7 (37.2)	177.9	0.2 (0.4)	1.8
2010	66.3	518.0	10.1 (15.2)	78.7	0.8 (1.2)	6.3	0.1 (0.1)	0.5	22.9 (34.6)	179.0	0.6 (0.9)	4.6
Avg. in 2008-2010	64.5	505.0	10.2 (15.8)	79.9	0.8 (1.2)	6.2	0.1 (0.2)	0.8	22.9 (35.6)	179.6	0.4 (0.5)	2.8
2011	70.8	553.8	9.9 (13.9)	77.2	1.3 (1.8)	9.9	1.0 (1.4)	8.0	22.4 (31.6)	174.9	0.7 (1.0)	5.5
2012	77.4	606.8	10.6 (13.7)	83.3	1.3 (1.7)	10.2	1.0 (1.3)	7.9	23.9 (30.9)	187.4	0.7 (1.0)	5.8
2013	88.1	692.3	11.8 (13.3)	92.4	1.5 (1.7)	11.6	1.1 (1.2)	8.6	27.9 (31.7)	219.3	0.8 (0.9)	6.2
2014	90.8	714.6	12.4 (13.6)	97.4	1.4 (1.5)	10.9	1.0 (1.2)	8.2	29.0 (31.9)	228.0	0.8 (0.8)	5.9
2015	91.7	721.8	12.6 (13.7)	99.0	1.3 (1.5)	10.5	1.1 (1.2)	8.4	29.6 (32.3)	233.1	0.7 (0.8)	5.5
2016	93.4	735.6	12.6 (13.5)	99.5	1.4 (1.5)	10.7	1.1 (1.1)	8.4	30.6 (32.8)	241.4	0.7 (0.8)	5.7
2017	99.6	786.2	13.4 (13.5)	105.8	1.4 (1.4)	11.3	1.1 (1.1)	8.7	32.6 (32.8)	257.6	0.8 (0.8)	6.1
2018	103.3	816.7	13.3 (12.9)	105.1	1.6 (1.6)	12.8	1.1 (1.1)	9.1	33.1 (32.0)	261.7	0.8 (0.8)	6.3
2019	102.2	809.9	12.4 (12.2)	98.5	1.5 (1.5)	11.9	1.2 (1.1)	9.2	32.4 (31.7)	256.4	0.8 (0.7)	6.0
2020	76.9	609.6	8.7 (11.4)	69.3	1.1 (1.5)	9.0	1.1 (1.5)	9.0	24.4 (31.7)	193.5	0.5 (0.7)	4.3
2021	96.0	765.3	12.0 (12.5)	95.9	1.4 (1.5)	11.4	1.2 (1.3)	9.7	31.0 (32.3)	247.4	0.7 (0.7)	5.2
**Year**	**Medication (1 billion yen), n, (%)**	**Medication per capita (yen)**	**Injection (1 billion yen), n, (%)**	**Injection per capita (yen)**	**Rehabilitation (1 billion yen), n, (%)**	**Rehabilitation per capita (yen)**	**Psychiatric specialty therapy (1 billion yen), n, (%)**	**Psychiatric specialty therapy per capita (yen)**	**Treatment (1 billion yen), n, (%)**	**Treatment per capita (yen)**	**Surgery (1 billion yen), n, (%)**	**Surgery per capita (yen)**
2008	7.7 (11.6)	60.1	0.3 (0.5)	2.4	0.0 (0.0)	0.2	0.0 (0.0)	0.0	0.5 (0.7)	3.7	16.5 (24.9)	129.1
2009	7.4 (12.0)	57.7	0.4 (0.6)	2.9	0.0 (0.1)	0.4	0.0 (0.0)	0.1	0.6 (1.0)	5.0	13.5 (22.1)	105.7
2010	6.7 (10.1)	52.5	1.5 (2.3)	11.7	0.1 (0.1)	0.5	0.0 (0.0)	0.1	0.5 (0.8)	3.9	17.0 (25.7)	133.1
Avg. in 2008-2010	7.3 (11.3)	56.8	0.7 (1.1)	5.6	0.0 (0.1)	0.4	0.0 (0.0)	0.1	0.5 (0.8)	4.2	15.7 (24.2)	122.6
2011	7.8 (11.0)	60.7	2.2 (3.1)	17.0	0.3 (0.4)	2.5	0.0 (0.1)	0.4	1.3 (1.9)	10.4	15.9 (22.5)	124.7
2012	7.8 (10.1)	61.3	2.8 (3.6)	22.1	0.3 (0.4)	2.7	0.1 (0.1)	0.4	1.3 (1.7)	10.3	18.0 (23.3)	141.4
2013	9.0 (10.2)	70.5	3.7 (4.1)	28.7	0.4 (0.5)	3.2	0.1 (0.1)	0.5	1.4 (1.6)	11.2	20.9 (23.8)	164.6
2014	9.0 (10.0)	71.2	5.2 (5.7)	40.8	0.4 (0.4)	3.2	0.1 (0.1)	0.5	1.4 (1.6)	11.3	15.8 (17.4)	124.3
2015	9.0 (9.8)	70.9	5.7 (6.3)	45.2	0.4 (0.4)	2.8	0.1 (0.1)	0.5	1.4 (1.5)	10.7	15.6 (17.0)	122.8
2016	8.9 (9.6)	70.3	5.9 (6.3)	46.7	0.4 (0.4)	2.9	0.1 (0.1)	0.4	1.3 (1.4)	10.6	16.8 (18.0)	132.4
2017	9.4 (9.4)	74.0	7.0 (7.0)	55.3	0.4 (0.4)	3.0	0.1 (0.1)	0.5	1.4 (1.4)	11.1	17.9 (18.0)	141.6
2018	9.2 (8.9)	72.4	8.0 (7.7)	63.3	0.4 (0.4)	3.1	0.1 (0.1)	0.5	1.4 (1.4)	11.1	23.6 (22.8)	186.6
2019	8.8 (8.6)	69.7	8.8 (8.6)	69.5	0.4 (0.3)	2.8	0.1 (0.1)	0.5	1.4 (1.3)	10.9	24.1 (23.5)	190.6
2020	7.6 (9.9)	60.1	8.2 (10.7)	64.9	0.3 (0.4)	2.5	0.1 (0.1)	0.4	1.2 (1.6)	9.5	16.4 (21.4)	130.3
2021	7.9 (8.2)	63.1	9.3 (9.7)	74.0	0.3 (0.3)	2.7	0.1 (0.1)	0.4	1.3 (1.3)	10.0	21.8 (22.7)	173.8
**Year**	**Anesthesia (1 billion yen), n, (%)**	**Anesthesia per capita (yen)**	**Radiotherapy (1 billion yen), n, (%)**	**Radiotherapy per capita (yen)**	**Pathological diagnosis (1 billion yen), n, (%)**	**Pathological diagnosis per capita (yen)**	**Hospitalization fee (1 billion yen), n, (%)**	**Hospitalization fee per capita (yen)**	**DPC/PDPS (1 billion yen), n, (%)**	**DPC/PDPS per capita (yen)**		
2008	0.2 (0.4)	1.9	-	-	0.0 (0.03)	0.1	3.5 (5.2)	27.2	2.6 (4.0)	20.6		
2009	0.3 (0.4)	2.1	-	-	0.0 (0.01)	0.1	1.7 (2.8)	13.3	3.3 (5.4)	26.0		
2010	0.3 (0.5)	2.5	-	-	0.0 (0.03)	0.2	1.7 (2.6)	13.6	3.9 (5.9)	30.8		
Avg. in 2008-2010	0.3 (0.4)	2.2	-	-	0.0 (0.02)	0.1	2.3 (3.6)	18.0	3.3 (5.1)	25.8		
2011	0.3 (0.5)	2.5	0.0 (0.03)	0.2	0.1 (0.09)	0.5	3.7 (5.3)	29.1	3.9 (5.5)	30.4		
2012	0.4 (0.5)	3.1	0.0 (0.03)	0.2	0.0 (0.06)	0.4	3.7 (4.7)	28.8	5.3 (6.8)	41.3		
2013	0.4 (0.5)	3.3	0.0 (0.03)	0.2	0.0 (0.05)	0.4	3.8 (4.3)	29.9	5.3 (6.0)	41.7		
2014	0.3 (0.4)	2.6	0.0 (0.03)	0.2	0.0 (0.05)	0.4	11.4 (12.6)	89.7	2.6 (2.8)	20.1		
2015	0.3 (0.3)	2.4	0.0 (0.03)	0.2	0.0 (0.05)	0.3	11.4 (12.4)	89.4	2.5 (2.8)	20.1		
2016	0.3 (0.3)	2.5	0.0 (0.03)	0.2	0.0 (0.05)	0.3	10.5 (11.3)	82.8	2.6 (2.8)	20.8		
2017	0.4 (0.4)	2.8	0.0 (0.03)	0.2	0.0 (0.05)	0.4	10.9 (11.0)	86.2	2.7 (2.7)	21.5		
2018	0.4 (0.4)	3.3	0.0 (0.03)	0.2	0.0 (0.05)	0.4	5.4 (5.2)	42.8	4.8 (4.6)	38.0		
2019	0.4 (0.4)	3.3	0.0 (0.03)	0.2	0.0 (0.05)	0.4	5.4 (5.3)	42.7	4.7 (4.6)	37.1		
2020	0.3 (0.4)	2.2	0.0 (0.03)	0.2	0.0 (0.04)	0.2	4.0 (5.1)	31.4	2.9 (3.7)	22.8		
2021	0.4 (0.4)	3.0	0.0 (0.03)	0.2	0.0 (0.04)	0.3	4.7 (4.9)	37.2	3.9 (4.1)	31.0		

Avg.: average; DPC/PDPS: Diagnosis Procedure Combination/Per-Diem Payment System.

### Expenses and number of calculations for ocular examinations in June

As shown in Table 1 and Figure 1, the proportion of total examination expenses to total medical expenses in 2021 was 10.0%. This was the fourth highest rate, followed by hospitalization fees (19.0%), DPC/PDPS (15.1%), and surgery (10.3%). The subsequent expenses were medication (6.9%), injections (6.9%), initial and repeat consultation (6.6%), treatment (5.5%), medical management (5.0%), at-home treatment (4.0%), diagnostic imaging (3.7%), rehabilitation (3.7%), anesthesia (1.3%), psychiatric specialty therapy (1.2%), radiotherapy (0.4%), and pathological diagnosis (0.4%).

As shown in Table 2 and Figure 2, the examination expenses for ophthalmic diseases accounted for 32.3% of ocular medical expenses in 2021. The subsequent expenses were surgery (22.7%), initial and repeat consultation fee (12.5%), injections (9.7%), medication (8.2%), hospitalization fee (4.9%), DPC/PDPS (4.1%), medical management (1.5%), treatment (1.3%), at-home treatment (1.3%), diagnostic imaging (0.7%), anesthesia (0.4%), rehabilitation (0.3%), psychiatric specialty therapy (0.1%), pathological diagnosis (0.04%), and radiotherapy (0.03%).

**Figure 3.**
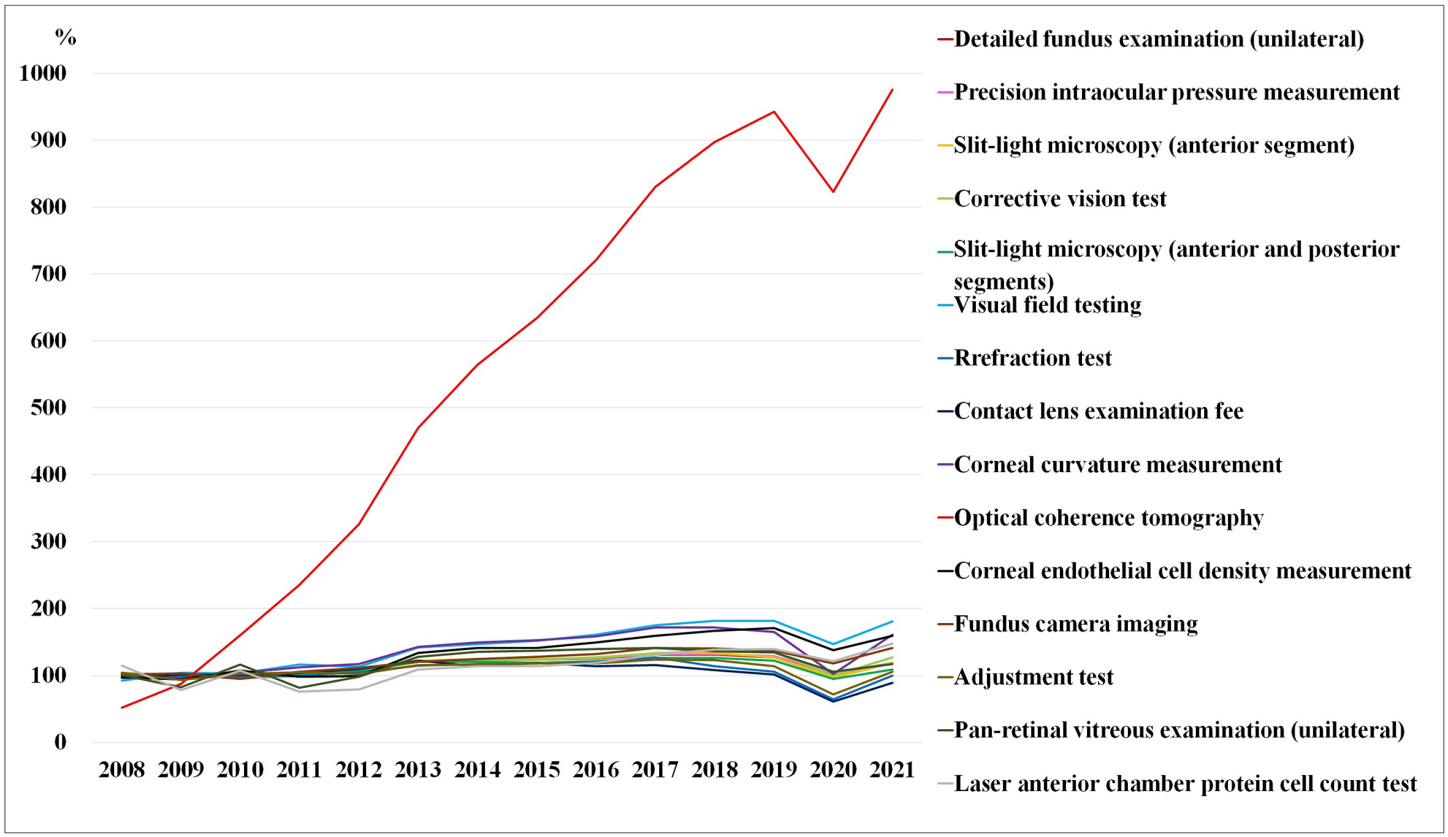
Trends in the expenses for the main ocular examinations, expressed as a percentage of the 2008-2010 average.

**Figure 4.**
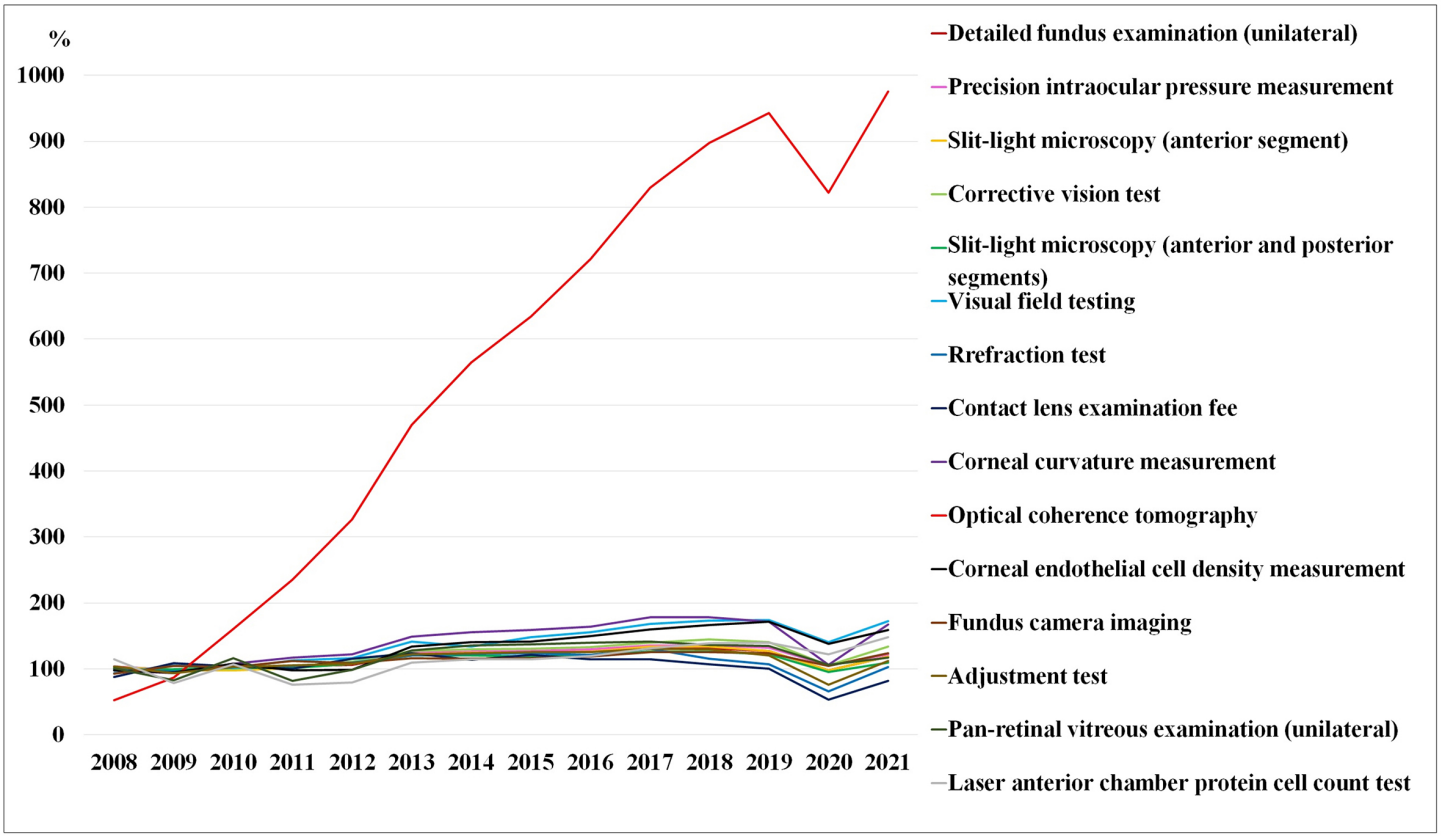
Trends in the numbers of the main ocular examinations, expressed as a percentage of the 2008-2010 average.

Table 3 and Figure 3 show the trends in expenses for the main ocular examinations, whereas Table 4 and Figure 4 demonstrate the trends in the number of main ocular examinations focused on the subclassification category from the MCAPHI, including ocular examination orders from non-ophthalmologists.

**Table 3.** Trends in the Expenses for the Main Ocular Examinations.

Year	2008	2009	2010	Avg. in 2008-2010	2011	2012	2013	2014	2015	2016	2017	2018	2019	2020	2021	2021/(Avg. in 2008-2010) (%)
Overall examinations	154.3	156.1	155.3	155.2	171.9	179.2	202.8	203.5	202.3	208.0	217.4	222.1	222.0	171.9	231.5	149.1
Ocular examination subtotal	24.6	24.1	24.2	24.3	25.4	26.9	31.1	31.9	32.3	33.2	35.1	35.4	34.6	26.4	32.8	135.0
Detailed fundus examination (unilateral)	5.68	5.35	5.38	5.47	5.66	5.81	6.65	6.76	6.77	6.89	7.20	7.18	6.95	5.33	6.52	119.1
Precision intraocular pressure measurement	4.27	4.06	3.93	4.09	4.13	4.37	4.96	5.05	5.07	5.16	5.39	5.40	5.26	4.20	4.86	118.8
Slit-light microscopy (anterior segment)	3.24	3.10	3.08	3.14	3.18	3.40	3.83	3.91	3.92	3.97	4.20	4.20	3.99	3.06	3.73	118.8
Corrective vision test	3.26	3.13	2.99	3.13	3.10	3.31	3.78	3.86	3.89	3.98	4.17	4.32	4.20	3.14	3.98	127.4
Slit-light microscopy (anterior and posterior segments)	1.65	1.66	1.70	1.67	1.69	1.80	2.01	2.02	1.99	2.03	2.11	2.11	2.05	1.59	1.83	109.3
Visual field testing	1.51	1.70	1.68	1.63	1.90	1.84	2.32	2.40	2.48	2.63	2.85	2.96	2.97	2.39	2.95	181.2
Refraction test	1.55	1.49	1.48	1.51	1.51	1.58	1.73	1.77	1.78	1.80	1.91	1.71	1.60	0.97	1.51	100.1
Contact lens examination fee	1.28	1.34	1.34	1.32	1.38	1.45	1.62	1.50	1.57	1.50	1.53	1.43	1.34	0.81	1.18	89.1
Corneal curvature measurement	0.88	0.84	0.92	0.88	0.99	1.04	1.26	1.32	1.35	1.39	1.51	1.51	1.46	0.90	1.42	161.0
Optical coherence tomography	0.15	0.26	0.47	0.30	0.70	0.96	1.39	1.67	1.88	2.13	2.45	2.65	2.79	2.43	2.88	975.6
Corneal endothelial cell density measurement	0.28	0.27	0.31	0.29	0.28	0.29	0.39	0.41	0.41	0.43	0.46	0.48	0.50	0.40	0.46	159.3
Fundus camera imaging	0.27	0.27	0.25	0.26	0.28	0.29	0.32	0.33	0.33	0.34	0.37	0.37	0.36	0.31	0.37	141.2
Adjustment test	0.23	0.21	0.23	0.22	0.23	0.23	0.26	0.26	0.26	0.26	0.28	0.27	0.25	0.16	0.24	105.7
Pan-retinal vitreous examination (unilateral)	0.12	0.10	0.14	0.12	0.10	0.12	0.15	0.16	0.17	0.17	0.17	0.16	0.16	0.13	0.14	117.1
Laser anterior chamber protein cell count test (1 billion yen)	0.07	0.05	0.07	0.06	0.05	0.05	0.07	0.07	0.07	0.08	0.08	0.09	0.09	0.08	0.10	148.1

Avg.: average.

**Table 4.** Trends in the number of main ocular examinations.

Year	2008	2009	2010	Avg. in 2008-2010	2011	2012	2013	2014	2015	2016	2017	2018	2019	2020	2021	2021/(Avg. in 2008-2010) (%)
Overall examinations	154.3	153.1	150.9	152.8	167.3	170.2	191.4	191.1	189.8	193.0	202.0	203.5	201.0	159.8	189.5	124.1
Ocular examination subtotal	33.8	32.7	33.3	33.3	34.7	36.7	41.8	42.6	42.9	43.7	46.1	46.1	44.5	33.5	41.9	125.8
Detailed fundus examination (unilateral)	10.14	9.55	9.61	9.77	10.11	10.38	11.87	12.07	12.09	12.31	12.86	12.82	12.40	9.52	11.64	119.1
Precision intraocular pressure measurement	5.02	4.78	4.78	4.86	5.03	5.33	6.05	6.16	6.18	6.29	6.57	6.58	6.41	5.11	5.92	121.9
Slit-light microscopy (anterior segment)	6.75	6.45	6.43	6.54	6.63	7.08	7.98	8.14	8.17	8.26	8.75	8.76	8.31	6.38	7.77	118.8
Corrective vision test	4.40	4.23	4.33	4.32	4.49	4.80	5.48	5.59	5.63	5.76	6.04	6.25	6.09	4.55	5.77	133.6
Slit-light microscopy (anterior and posterior segments)	1.48	1.48	1.52	1.49	1.51	1.61	1.80	1.80	1.78	1.81	1.89	1.89	1.83	1.42	1.63	109.3
Visual field testing	0.61	0.69	0.66	0.65	0.74	0.75	0.92	0.87	0.96	1.01	1.09	1.13	1.13	0.91	1.12	172.2
Refraction test	2.09	2.01	2.15	2.08	2.19	2.29	2.51	2.56	2.58	2.53	2.73	2.40	2.24	1.37	2.14	102.7
Contact lens examination fee	0.93	1.15	1.10	1.06	1.06	1.22	1.31	1.20	1.28	1.21	1.21	1.13	1.06	0.56	0.86	81.7
Corneal curvature measurement	0.99	0.95	1.09	1.01	1.18	1.23	1.50	1.57	1.61	1.66	1.80	1.80	1.73	1.07	1.69	167.2
Optical coherence tomography	0.08	0.13	0.24	0.15	0.35	0.48	0.70	0.83	0.94	1.07	1.23	1.33	1.39	1.22	1.44	975.6
Corneal endothelial cell density measurement	0.18	0.17	0.19	0.18	0.18	0.18	0.24	0.26	0.26	0.27	0.29	0.30	0.31	0.25	0.29	159.3
Fundus camera imaging	0.29	0.33	0.32	0.31	0.35	0.34	0.36	0.36	0.37	0.37	0.39	0.39	0.39	0.33	0.39	123.9
Adjustment test	0.31	0.28	0.31	0.30	0.32	0.33	0.37	0.37	0.38	0.38	0.39	0.39	0.36	0.23	0.34	111.8
Pan-retinal vitreous examination (unilateral)	0.08	0.07	0.09	0.08	0.07	0.08	0.10	0.11	0.11	0.11	0.11	0.11	0.11	0.08	0.09	117.1
Laser anterior chamber protein cell count test (1 million times)	0.05	0.03	0.04	0.04	0.03	0.03	0.04	0.05	0.05	0.05	0.05	0.06	0.06	0.05	0.06	148.1

Trends in the top 15 items, on average, from 2008 to 2010 are presented because they accounted for approximately 98% of the overall ocular examination.

The total examination and ocular examination subtotal expenses increased from 155.2 billion yen to 231.5 billion yen and from 24.3 billion yen to 32.8 billion yen over 14 years, respectively. optical coherence tomography (OCT) increased by 875.6%, visual field testing increased by 81.2%, corneal curvature measurement increased by 61.0%, corneal endothelial cell density measurement increased by 59.3%, laser anterior chamber protein cell count test increased by 48.1%, and fundus camera imaging increased by 41.2%, corrective vision tests increased by 27.4%, detailed fundus examination (unilateral) increased by 19.1%, precision intraocular pressure measurement increased by 18.8%, slit-lamp microscopy (anterior segment) increased by 18.8%, pan-retinal vitreous examination (unilateral) increased by 17.1%, slit-lamp microscopy (anterior and posterior segments) increased by 9.3%, and adjustment test increased by 5.7%. The refraction test results remained unchanged for more than 14 years. Only the CL examination fee expenses decreased by 10.9% for 15 items (Table 3 and Figure 3).

In particular, among the examinations newly established after 2009, OCT angiography, established in 2018, increased from 81.7 million yen (20,419 times) in 2018 to 173.8 million yen (43,437 times) in 2021. Optical axial length measurement established in 2010, increased from 21.0 million yen (14,006 times) in 2010 to 88.3 million yen (58,881 times) in 2021. The contrast sensitivity test, established in 2018, increased from 20.5 million yen (9,922 times) in 2018 to 40.5 million yen (19,552 times) in 2021. Anterior segment OCT analysis, established in 2018, increased from 29.1 million yen (10,989 times) in 2018 to 31.7 million yen (11,973 times) in 2021.

### COVID-19 pandemic impact

The COVID-19 pandemic, which began in February 2020, decreased medical expenses from 2,360 billion yen in 2019 to 2,033 billion yen (a decrease of 13.8%) in June 2020 and 2,307 billion yen (a decrease of 2.3%) in June 2021. Ocular medical expenses decreased by 24.7% in 2020 and by 6.0% in 2021 compared to 2019. Compared to the average for the fiscal years 2017 to 2019, the total examination expenses decreased by 22.0% in 2020, whereas ocular examination expenses decreased by 24.8%, significantly impacting ocular examination expenses. The refraction test was the most affected examination item, with a loss of 44.3%. The CL examination fee decreased by 43.3%, the adjustment test decreased by 40.0%, and the corneal curvature measurement decreased by 39.8%.

## Discussion

This study provides new findings on ocular examinations in Japan.

The MHLW publishes two types of medical expense databases: the ENMCE and MCAPHI. The ENMCE provides an estimate of the expenses required to treat injuries and illnesses covered by insurance medical care at medical institutions. Although it allows for an accurate understanding of the total medical expenses, it does not provide detailed medical procedure categories. Therefore, we acquired the MCAPHI data for detailed ocular examination expenses.

The MCAPHI compiles receipt data issued by insurance medical institutions and publishes details of medical procedures for investigating detailed trends in each medical field; however, the government only collects data in June. Consequently, a possibility of discrepancies exists in annual ENMCE data.

### Expenses and number of ocular examinations in June

Total medical expenses at medical facilities increased by 35.1%, and medical expenses for ophthalmic diseases increased by 49.0% over 14 years. This is attributed to the surge in ophthalmic injections (e.g., anti-vascular endothelial growth factor agents ^(1)^) by about 13 times (total medical expenses increased by 1.92 times).

In 2021, examination costs accounted for the highest proportion of total ophthalmic medical care expenses, whereas hospitalization costs accounted for the largest proportion of total medical expenses. This is ascribed to annual outpatient expenses for ophthalmology that are approximately three times higher than inpatient expenses ^(10)^.

In June 2021, the proportion of inpatient examination expenses in medical expenses for medical treatment was 8.3%. However, inpatient examination expenses for ophthalmic diseases accounted for only 0.8%. Low inpatient medical expenses in ophthalmology are largely due to the recent spread of same-day surgery, which may have decreased hospitalization fees ^(1)^.

The number of total examinations has increased by 49.1% over 14 years, whereas ocular examination expenses witnessed an increase of only 35.0%. This may be due to the revision of ophthalmology medical fees, which were adjusted based on data such as the price of examination equipment and the time required, and included a reduction in medical fee points ^(12)^.

The OCT score (200 points) has not been revised for 14 years ^(12)^. The number of examinations and expenses increased the most, by 875.6%. As far as the author has researched, there are very few research reports that provide figures for the prevalence of OCT. OCT is a tomographic imaging technique that uses weak near-infrared light to visualize the internal structure of scattering bodies such as living organisms. Because OCT was commercially released in 1996, it has been able to examine the retinal layer noninvasively and has replaced conventional fundus cameras. OCT is able to diagnose lesions more accurately than ophthalmoscopic observation. OCT images also provide information on the thickness of retinal layer structures. Understanding changes in the disease and the effectiveness of treatment requires this test, which is essential ^(13)^. In Japan, it was covered by insurance in April 2008 and is widely used not only in core hospitals but also in clinics. It has become popular in recent years because of its effectiveness in diagnosing retinal diseases ^(14)^. OCT has continued to develop, becoming faster, more extensive, more functional, and simpler ^(14)^. This examination is useful for the early detection and treatment of eye diseases such as glaucoma, age-related macular degeneration, and diabetic retinopathy.

**Figure 5.**
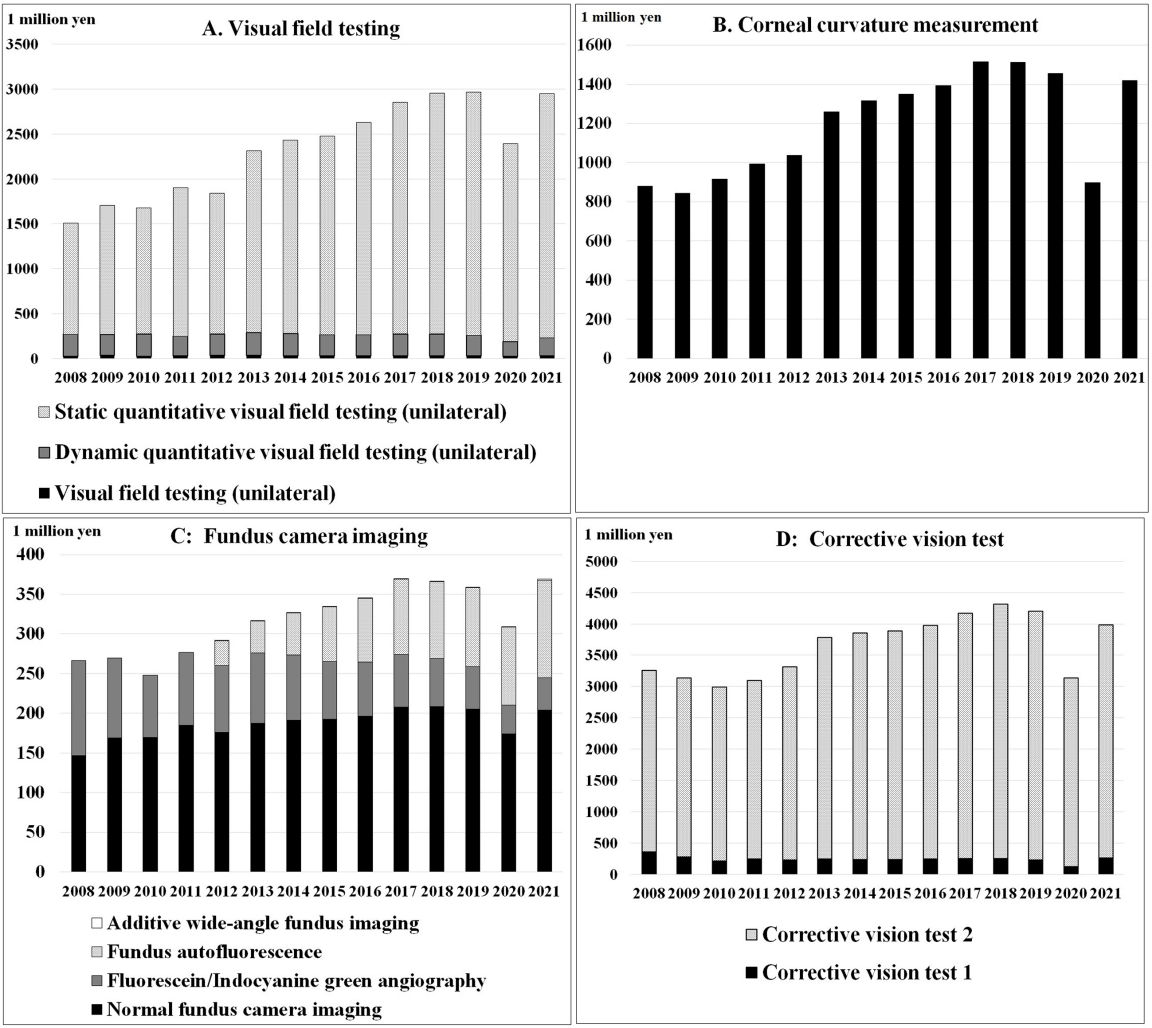
Trends in the main ocular examinations due to revisions to medical fees (Visual field testing to corrective vision test).

**Figure 6.**
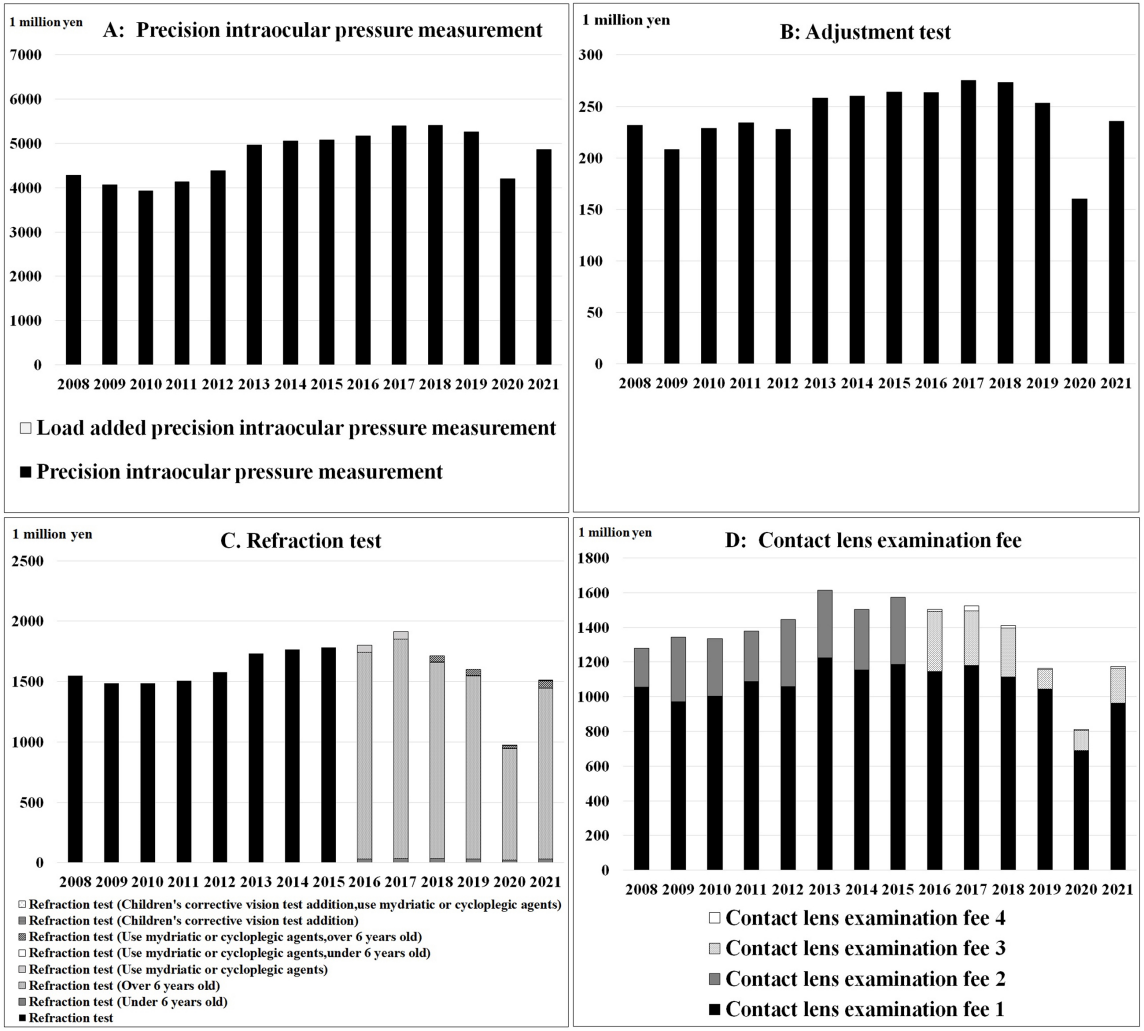
Trends in the main ocular examinations due to revisions to medical fees (Precision intraocular pressure measurement to contact lens examination fee).

Figures 5 and 6 show the eight examinations for which the evaluation of the examination fee changed due to the main revision of the examination fee.

The visual field testing fees for the precision visual field test (unilateral) (38 points) and dynamic quantitative visual field test (unilateral) (195 points) were not revised, and the fee for the static quantitative visual field test (unilateral) (300 points) was reduced by 10 points in 2012 ^(12)^. This had a temporary effect; however, expenses increased by 81.2% (Figure 5A). The usage likely increased with the number of patients with glaucoma ^(15)^.

Corneal curvature measurement expenses decreased from 89 to 84 points in 2010; however, expenses increased (Figure 5B) ^(12)^. This examination is needed in all situations, such as vision tests and determining the strength of the surgery ^(16)^.

Corneal endothelial cell density measurement (160 points) was not revised after 14 years ^(12)^. The frequency of use increased simultaneously with the increase in cataract surgery, which has become particularly popular because it is used before and after surgery ^(16)^.

The laser anterior chamber protein cell count test result (160 points) was not revised after 14 years of follow-up ^(12)^. It measures protein concentration and cell count in the anterior chamber to diagnose the degree of anterior eye inflammation before and after cataract surgery and uveitis. The expenses of surgery in 2021 increased by 48.0% compared to the average from 2007 to 2009 ^(1)^. Therefore, this increase may have been influenced by an increase in the number of surgeries.

Fundus camera imaging expenses did not increase considerably as the number of examinations, which could be attributed to a revision in examination fees. In 2012, fundus autofluorescence (510 points) and wide-angle fundus imaging (100 points) were added, which allowed observation of the fluorescence emitted by the fundus itself without using a contrast medium (Figure 5C) ^(12)^. In 2016, fundus autofluorescence exceeded fluorescein/indocyanine green angiography to reach 122.4 million yen in 2021, and fluorescein/indocyanine green angiography was 40.1 million yen in 2021.

Corrective vision test expenses increased by 27.4%; however, the number of examinations increased by 33.6%, which could be ascribed to a reduction in examination fees from 74 to 69 points in 2010 (Figure 5D) ^(12)^.

Precision intraocular pressure measurement expenses increased by 18.8%; however, the number of examinations increased by 21.9%, which may have been due to the impact of the reduction in examination fees from 85 to 82 points in 2010 (Figure 6A) ^(12)^.

The examination fee for detailed fundus examination (unilateral) (56 points), slit-light microscopy (anterior segment) (48 points), pan-retinal vitreous examination (unilateral) (150 points), and slit-light microscopy (anterior and posterior segments) (112 points) did not undergo revision in 14 years ^(12)^. All these are basic ocular examinations; however, the increase may be due to an increase in the number of initial and repeat consultation fees for ocular diseases, which increased by 17.3% over 14 years.

Although adjustment test expenses increased by 5.7%, the number of tests increased by 11.8%, which may be due to a reduction from 74 to 70 points in 2012 (Figure 6B) ^(12)^.

The refraction test score was reduced from 74 points to 69 points in 2010, and the target age and mydriatic drugs were considered in 2016; however, the expenses did not change (Figure 6C) ^(12)^. Ametropia refers to myopia, hyperopia, and astigmatism. The use of refraction testing for people aged 6 and over has declined since 2018, so needs in the field may be changing.

Only the CL examination fee expenses decreased over 14 years. In 2016, the 2008 CL examination fees 1 (200 points) and 2 (56 points) were changed to CL examination fees 1 (200 points) and 3 (56 points), and new CL examination fees 2 (180 points) and 4 (50 points) were added (Figure 6D) ^(12)^. Specifically, the following has been added to the existing facility criteria for CL examination fees 1: for insurance medical institutions that do not have inpatient beds, the number of patients for whom CL examination fees are calculated is less than 10,000 per year, or the CL payment rate at the facility is less than 95% (*) ^(12)^.

CL examination fee 2 is a facility that meets CL examination fee 1 but does not meet (*). The CL examination fee 3 is a health insurance medical institution that does not meet the existing facility criteria for CL examination fee 1 and meets (*). The CL examination fee 4 is not met. CL examination fee expenses may have decreased since 2017 because of the revision of the examination fee assessment.

According to the Japan Contact Lens Association, the shipment value of CL and CL care products increased from 243.3 billion yen in 2014 to 334 billion yen in 2023 ^(17)^. However, the percentage of women (15-29 years old) who visited ophthalmologists every time they purchased CL decreased from 60% in 2014 to 20% in 2022 ^(18), (19)^. CL examination fee expenses decreased in 14 years, possibly because of a decline in the number of patients visiting ophthalmologists.

Among the examinations newly established after 2009, the expenses of OCT angiography (400 points) surpassed pan-retinal vitreous examination (unilateral) and the laser anterior chamber protein cell count test in 2021. However, the number of tests was not high, which may be due to the medical fee points (400 points). This examination uses OCT to three-dimensionally evaluate the condition of blood vessels in the retina and macula and is ascribed to increasing use instead of fluorescein fundus angiography, requiring the instillation of a contrast agent, which increased by 2.13 times from 2018 to 2021.

Optical axial length measurement (150 points) is a necessary examination before cataract surgery to measure the axis of the ocular lens and determine the strength of the intraocular lens to be inserted into the eyes, which increased by 4.2 times from 2010 to 2021. This increase may have been influenced by an increase in the number of surgeries ^(1)^.

The contrast sensitivity test (207 points) is different from a vision test that measures the ability to recognize a normal vision chart in that it distinguishes between light and dark and the shade of color. It can precisely examine changes in a patient’s vision that cannot be detected by a vision test and is lower for cataracts, glaucoma, and diabetic retinopathy. This expense increased by 1.97 times from 2018 to 2021.

Anterior segment OCT analysis (265 points) allows the observation of cross-sectional surfaces of the cornea, angle, and iris, and numerical analysis of three-dimensional structures, which cannot be performed with conventional ocular examinations ^(20)^. This test allows numerical analysis, including irregular astigmatism. This expense increased by 1.09 times from 2018 to 2021.

### COVID-19 pandemic impact

COVID-19 has had a major impact on Japan, with ocular examination expenses decreasing more than the national examination expenses, and the impact on ocular examination expenses being greater. The refraction and adjustment test (which have not witnessed significant increases for 14 years), the CL examination fee, and corneal curvature measurement were particularly affected, possibly because of declines in patients visiting ophthalmologists and surgeries due to COVID-19 ^(1)^. In addition to known ocular surgeries and outpatient consultation behaviors ^(1)^, ocular examinations were suppressed.

### Research limitations

The following limitations should be noted before interpreting the results. This study used government data; therefore, the results depend on the accuracy of the government survey. Insurance coverage and classification of medical procedures were provided by the MHLW. A possibility of clerical errors during the classification exists. Second, the MCAPHI dataset contains June data and not annual data, resulting in a possibility of errors in each medical service. In 2021, the total and ocular medical expenses in June from the MCAPHI reached 2.31 trillion yen and 96.0 billion yen, respectively. We multiplied these expenses by 12; the estimated annual nationwide and ocular expenses were 27.7 trillion yen and 1,152 billion yen, respectively. The actual expenses annually were 32.4 trillion yen and 1,163 billion yen, respectively. Fewer cases of seasonal infections and influenza in June possibly caused this difference ^(1)^. Thirdly, the category of ‘medical procedures (subclassification)’ does not correspond to medical departments or injury and disease classifications, including examination orders from non-ophthalmologists.

### Conclusion

The total and ocular medical expenses increased from 2008 to 2021. In terms of examination expenses, nationwide examinations have increased by 49.1%, whereas ocular examinations have increased by only 35.0%. Examination expenses account for the largest proportion of ophthalmology treatments. OCT use increased by 875.6% over 14 years. Visual field testing, corneal curvature measurement, and corneal endothelial cell density measurements increased by 81.2 %, 61.0 %, and 59.3% in 14 years, respectively. Only CL examination fee expenses decreased over the 14 years, possibly because of a decline in the number of patients visiting ophthalmologists. Ocular examination expenses were more affected by the COVID-19 pandemic than were the total medical expenses. This study suggests possible influences, including advances in testing equipment, changes in patient behavior, and revisions to medical fees. Altogether, these findings may be beneficial for selecting examination items and allocating medical resources.

## Article Information

### Acknowledgement

The author would like to thank Editage (www.editage.jp) for the English language editing. The author appreciates the Ministry of Health, Labour and Welfare for publishing valuable open data.

### Author Contributions

Tomoko Kanaya: study concept and design, data collection, data analysis, and manuscript writing and editing.

### Conflicts of Interest

None

### IRB Approval Code and Name of the Institution

Only open data sources were used in this study; ethical review and approval was not required.
